# Composition of Slow-Growing Male Chicken’s Meat and Bone Quality as Affected by Dietary *Moringa oleifera* Lam. Meal

**DOI:** 10.3390/ani12243482

**Published:** 2022-12-09

**Authors:** Esther Faustin-Evaris, Luis A. Sarmiento-Franco, Concepción M. Capetillo-Leal, Carlos A. Sandoval-Castro

**Affiliations:** Faculty of Veterinary Medicine and Animal Science, University of Yucatan (UADY), km 15.5 Carretera Mérida-Xmatkuil, Mérida 97315, Yucatán, Mexico

**Keywords:** slow-growing male chickens, Dominant CZ Blue D 107, meat composition, bone quality, outdoor access, moringa, tropical climate

## Abstract

**Simple Summary:**

Chicken meat is one of the most consumed in the world because of its nutritional composition, which is influenced by several factors including the genotype, sex, age, health status of the birds, and the production system used to raise them in. It has been demonstrated that diet and the possibility to exercise are two key components that have direct effects on the nutrient content of chicken meat. However, few studies have focused on including a vegetable material that is relatively inexpensive such as *Moringa oleifera* Lam. meal (MOM) to reach this goal while providing outdoor access to the birds. Therefore, this study explored the influence of different levels of MOM on the meat and bone quality of slow-growing male chickens raised with access to outdoors in the tropics. The results demonstrated that breast muscle ash content significantly increased with MOM, whereas meat dry matter, protein, and fat content remained unaffected by the diets. Oleic, linoleic, stearic, palmitic, and palmitoleic acids were present in both the breast and leg muscles. Bone quality parameters, such as femoral and tibial length, tibial diameter, bone ash, and phosphorous content, were significantly influenced by the diets in a positive way. It is concluded that MOM can be used to positively modify the bone quality of slow-growing layer-type male chickens raised with outdoor access in a tropical environment.

**Abstract:**

This study investigated the impact of *Moringa oleifera* Lam. meal (MOM) on meat nutritional properties and bone quality of slow-growing layer-type male chickens raised in semi-intensive conditions. A total of 198, 72-d-old Dominant Blue D 107 male chickens, with an average weight of 1093 ± 15.2 g, were randomly assigned to three dietary treatments supplemented with 0, 3, and 6% of MOM that corresponded to T1, T2, and T3, respectively. Each treatment, consisting of six replicated floor pens of 11 birds, had access to the outdoors for 49 days. The results showed that breast muscle ash percentage was significantly greater (*P* ≤ 0.05) in T2 in comparison to the T1 group. Meat dry matter, protein, and fat content were not influenced by the treatments (*P* > 0.05). Regardless of the treatments, oleic acid (C18:1N9C) was numerically more abundant in the breast than in the leg muscle. Alternatively, femoral and tibial lengths were shorter (*P* ≤ 0.05) in birds fed 3% MOM than the two other groups. Moreover, birds fed with MOM had greater tibial diameter (*P* ≤ 0.05) than those that were fed without MOM. In addition, bone ash content and phosphorous amount were significantly higher (*P* ≤ 0.05) in birds fed 6% MOM compared to those fed without MOM. The data of this study indicate that up to 6% of MOM may be added to the diet of slow-growing layer-type male chickens raised with outdoor access under tropical conditions to improve bone quality traits.

## 1. Introduction

Meat’s biochemical profile reveals its nutritive value. Chicken meat is an excellent source of macronutrients (protein and lipids) and micronutrients (vitamins and minerals) and a popular food item in most countries. According to the National Chicken Council [1], in the USA, the per capita consumption of chicken in 2020 was 44.36 lbs. Global chicken meat and processed chicken meat product consumption is expected to increase within the next decade as they are perceived to be healthy sources of animal protein with low fat and high polyunsaturated fatty acid (PUFA) content. Improving chicken meat’s nutritional value (high DM, protein, ash, and PUFA, but low-fat content) is essential to ensure that consumers have access to products of high-nutritious quality. It has been demonstrated that the rearing system or the living conditions of the chickens can influence their meat nutritive properties [2,3]. For instance, raw meat from chickens raised with access to the outdoors has contained more protein and ash, but less fat content than that derived from indoor-raised ones [4]. It has also been pointed out that the nutritional composition of chicken meat and its derivative products can be enhanced through nutritional management, particularly due to the provision of dietary bioactive components (i.e., proteins, fiber, vitamins, minerals, antioxidants, polyphenol) of plant-origin raw materials [5,6]. Thus, developing new feeding strategies to improve the nutritional value of chicken meat and ensure healthy end products is an increasingly important factor for nutritionists and producers, as consumers are more aware of their health and the well-being of the birds they consume.

Healthy bones promote healthy birds that might in turn have better meat nutritional properties. Among bone health indicators are bone length, weight, diameter, Seedor, and robusticity index, and ash content, with the latter measuring mineral content, particularly calcium and phosphorous percentage [4]. Birds with leg problems are not profitable to producers [7] and are an indication of impaired animal welfare. Leg complications, such as tibial dyschondroplasia, lameness, and foot pad dermatitis, can result from a deficiency of nutrients or lack of exercise. There is also a possible genetic effect, as fast-growing chickens tend to present more leg or skeletal problems than slow-growing ones [8,9]. Thus, chicken bone health can be affected by the dietary amount of minerals as well as the ability and possibility to exercise (walking, lying, standing, scratching, sand bathing, etc.) [4,10,11]. Therefore, elaborating feeding methods that can have a positive direct impact on the bird’s bone health is another critical element for nutritionists and producers to take into account, with consumers and advocates for animal welfare in mind.

On the other hand, slow-growing layer-type male chickens are a complete waste in the egg industry as they are generally killed at 1-day-old, even though they are proven to be well-adapted to production systems with outdoor access [4,12,13]. These birds may not produce enough meat to meet large-scale demand [14]; however, their meat can be used in the processed chicken meat product industry. The process of adding value to the meat of these birds may be an important step in encouraging producers to invest resources in this specific niche. This process can be achieved through the enhancement of the nutritional composition of the meat. Nevertheless, few studies have focused on including a vegetable material that is relatively inexpensive and sustainable, such as *Moringa oleifera* Lam. (MO) to reach this goal.

MO, a plant that is available in most tropical and subtropical countries, is rich in nutrients and bioactive compounds such as protein, fiber, minerals, vitamins, and antioxidants. For instance, one hundred grams of dry MO meal (MOM) contains a significant amount of minerals such as calcium (2185–3050 mg), potassium (1236–1384 mg), magnesium (86–448 mg), and phosphorus (204–252 mg); vitamins and antioxidants such as vitamin A (16.3–18.9 mg), vitamin B1 (2.02–2.6 mg), vitamin B2 (19.82–21.3 mg), vitamin B3 (7.6–8.3 mg), and vitamin C (15.8–17.3 mg); protein (29.4–40 g); fat (5.2–6.5 g); fiber (12.5–21.09 g); calories (329 cal); and carbohydrates (38–41.2 g) [15]. One of the most important antioxidants present in MO is vitamin E (10.8–77 mg/100 g of dry leaves), which is essential in protecting cell membranes from oxidative damage, preventing the meat from being oxidized and prolonging meat shelf life. Additionally, MO is characterized by its low omega-6 fatty acid content (7.64 g/100 g of dietary fat), high omega-3 fatty acid level (44.57 g/100 g of dietary fat), and low omega-6/omega-3 ratio (0.17 g/100 g of dietary fat) [16] that makes it a vegetable ingredient used to positively modify meat fatty acid profile in chicken [17,18,19], pork [20] and rabbit [21]. All these nutritional properties of MO suggest that its inclusion in chicken diets may have a beneficial impact on the chicken’s meat nutritional value and bone quality, depending on several other factors such as nutritional (inclusion rates), genetic (animal growth rate), environmental (tropical, dry, temperate climate), and the applied production system (with or without outdoor access). Nonetheless, the direct effect of MOM on meat chemical composition and bone quality of layer male chickens is yet to be evaluated in the tropics. Therefore, this investigation aimed to evaluate the impact of different levels of MOM on the meat and bone quality of slow-growing Dominant Blue D 107 male chickens raised with outdoor access in the tropics.

## 2. Materials and Methods

### 2.1. Location and Duration of the Experiment

This study was carried out at the poultry facilities of the Faculty of Veterinary Medicine and Animal Science (FMVZ) of the University of Yucatan (UADY) in Mexico for forty-nine days and followed all bioethics requirements of the Biological and Agricultural Sciences Campus (CCBA) of the UADY (CB-CCBA-S-2022). The climate of the region is subtropical warm subhumid, with an average annual temperature of 26 °C and an average annual rainfall of 1100 mm [22].

### 2.2. Birds and Management of the Experiment

A total of three hundred and six, 1-day-old layer-type slow-growing Dominant Blue D 107 chickens were purchased from a local commercial hatchery and arrived at the FMVZ. All birds were farmed in a single-spacious floor pen, where they received water and commercial feed (21% CP and 2.9 Mcal/kg ME) *ad libitum* until reaching 72 days of age. At 73 d-old, one hundred and ninety-eight male chickens with an average live weight of 1093 ± 127 g were individually identified with leg bands and randomly allocated to three treatments.

The experimental design was completely randomized and consisted of three dietary treatments that contained MOM supplemented at levels of 0, 3, and 6% that corresponded to T1 (control), T2, and T3 groups, respectively. Each treatment consisted of 6 replicates (floor pens), with each floor pen containing 11 birds. Chickens, housed in 18 floor pens (2.3 m × 1 m, each) equipped with shaving wood as bed and feeders and drinkers, received natural daylight and free access to experimental finisher diets and water. All birds had daily outdoor access to 18 pasture area courts (11.0 m × 1 m, each) via a doorway (50 cm × 70 cm, each) according to their group.

Fresh MO was collected from the FMVZ-UADY area and a nearby location. Leaves and edible green stems were dried at 60 °C for 72 h, ground (3 mm mesh), and added in proportions of 3 and 6% to the diets. Diets were formulated to contain 2.9 Mcal/kg metabolizable energy and 17% crude protein. The chemical composition of *Moringa oleifera* meal (MOM) is presented in Table 1; however, it is worth mentioning that the actual chemical composition of the diets revealed that the values for crude protein (CP) and crude fiber (CF) were different from the calculated values. The procedures of AOAC International [23] were used to analyze CP, ether extract (EE), and ash from MOM. CF (ANKOM method no. 7), neutral detergent fiber (ANKOM method no. 6), acid detergent fiber (ANKOM method no. 5), and lignin (ANKOM method no. 8) were analyzed according to Van Soest et al. [24]. Total phenols (Folin–Ciocalteu method), total tannins (adding polyvinyl polypyrrolidone), and condensed tannins (vanillin method) were measured as reported by Ortíz-Domínguez et al. [25]. Feed samples were analyzed using the methods of NMX [26] for dry matter, and AOAC International [23] for crude protein and fiber. The ingredients and nutrient composition of the experimental diets have been previously reported by Evaris et al. [14].

### 2.3. Meat Quality Evaluation

On day 120, the weight of each bird/replicate floor pen was registered, and all birds had access to water without feeding for 12 h before slaughtering. On day 121, four birds/replicate floor pens were randomly chosen and manually slaughtered by jugular incision and plucked in an automated machine. Carcasses were eviscerated, prechilled (4 °C for 1 h) and chilled (4 °C for 18 h) before sampling. Each sample consisted of 50-g meat without skin taken from each bird/replicate floor pen, for a total of 200 g/sample. Six samples/treatments for breast muscle and 6 samples/treatments for leg (drumstick and thigh) muscle were analyzed for meat macronutrient content (protein, fat, dry matter, and ash). The meat fatty acid profile was also determined. However, a preliminary assessment of expected differences in the fatty acid profile showed that a larger sample size than currently available from the experimental samples would have been needed in order to detect significant differences between treatments. Thus, due to the actual sample size and budgetary restrictions, a pooled sample per treatment was prepared for breast muscle and for leg muscle. The rationale for pooled samples was that it would allow us to describe the average qualitative changes in the fatty acid profile. This approach has been previously employed by Evaris et al. [4]. Each sample was homogenized through a blender and kept in a plastic bag at −20 °C until analysis.

Meat nutrient composition was determined using the following methods: MX [26] for DM, AOAC International [23] for CP, Thiex et al. [27] for EE, and NMX [28] for ash content. The fatty acid profile was determined by gas chromatography as described by Evaris et al. [4] following the methods of AOAC Official Method 996.06 (Analysis of Methyl Esters by Capilary GLC) and AOCS Official Method Ce 2–66 (Preparation of Methyl Esters of Fatty Acids) [23].

### 2.4. Bone Quality Assessment

A knife was used to manually remove the left femur and tibia bones from 24 birds per treatment (4 birds/replicate pen floor). An electric scale and a vernier were used to obtain the data on each bone’s weight, length, and diameter (from the bone’s mid-length). Bone samples were taken for laboratory analysis, with each sample consisting of 2 left femurs and 2 left tibiae for a total of 4 bones per sample. A total of 12 samples/treatments were used to analyze bone dry matter and ash content. The methods of NMX [26] and NMX [28] were used to determine DM and ash content, respectively. Thereafter, 1 sample of bone’s dry matter from each replicate was randomly selected to determine calcium and phosphorous content, resulting in 6 samples/treatments. Bone calcium and phosphorus were analyzed by titration [29] and colorimetric methods [30], respectively. Seedor and robusticity index, as well as bone relative density for each bone, were calculated through the following formulas: Seedor index = Weight/Length [31]; Robustness index = length/∛weight [32]; Bone relative density = bone weight/live body weight X 100 [4,33,34].

### 2.5. Statistical Analyses

The data for meat proximate composition and bone quality were analyzed by one-way ANOVA using the statistical software Minitab 19 [35]. The Tukey test was used to compare the means of treatments. Differences were considered statistically significant at *P* ≤ 0.05. Data are shown as the mean and standard error of means (SEM), and they are on a dry matter basis.

## 3. Results

### 3.1. Diet Effects on Meat Quality Characteristics

The effects of MOM dietary inclusion on meat nutrient composition are presented in Table 2. Compared to T1, MOM significantly increased (*P* ≤ 0.05) breast ash content in T2. Dry matter, protein, and fat content of breast and leg muscles were not significantly influenced by treatments (*P* > 0.05). Nonetheless, the transition from T1 to T2 or T3 revealed that relative numerical values for fat content in breast muscle decreased by 16.41% (2.62 vs. 2.19, T1 and T2, respectively) or by 14.89% (2.62 vs. 2.23, T1 vs. T3, respectively) but dry matter content in the same muscle increased by 0.91% (26.44 vs. 26.68, T1 and T2, respectively) or 0.98% (26.44 vs. 26.70, T1 vs. T3, respectively). Similarly, numerical values for the protein content of breast muscle increased with MOM. However, leg muscle relative numerical values for dry matter content revealed a 1.89% increase from T1 to T2 (25.35 vs. 25.83), but a 3.08% decrease from T1 to T3 (25.35 vs. 24.57). Between T1 and T2, there was a 1.24% reduction in leg muscle fat content (10.52 vs. 10.39). This decline even reached 21.29% (10.52 vs. 8.28) for birds fed T3 in comparison to those that were fed T1.

The effects of dietary treatments on meat fatty acid profile are shown in Table 3. In descending order, in terms of abundance, the fatty acid profile of the breast and the leg muscles revealed the presence of four major acids, which were oleic acid, palmitic acid, linoleic acid, and stearic acid. They accounted for 93.61% and 92.92% of breast and leg muscles, respectively. While stearic acid numerically increased by more than 30%, linoleic acid decreased by at least 5% in the breasts of the birds that were fed MOM in comparison to those that were fed without MOM. Palmitoleic acid was more abundant in the leg than in the breast muscle.

### 3.2. Diet Effects on Bone Quality Traits

Table 4 illustrates the effects of dietary treatments on some aspects of bone quality. The length of the femoral and tibial bones of T2 birds was reduced (*P* ≤ 0.05) by 2% in comparison to the other two treatments. There was a 4.95% decrease (*P = 0.021*) in the tibial diameter of birds fed without MOM. Compared to the birds from T3, leg bones’ ash content was significantly lowered (*P* ≤ 0.05) in birds from T1 by 5.43%. Bone phosphorous content was significantly higher with 6% MOM in comparison to 0%. The tibial robusticity index approached significance (*P = 0.055*) as it tended to decrease in birds fed with 3% MOM. Although statistical differences were not observed in leg bone dry matter and calcium content, they were numerically improved with increased MOM dietary inclusion. There were no effects of treatments on femoral and tibial weights, Seedor index, robusticity index, relative density, and femoral diameter; however, the numerical values were most likely to be lower in birds fed with T2.

## 4. Discussion

### 4.1. Influence of Moringa oleifera Lam. Meal (MOM) on Meat Nutritional Composition

Meat’s nutritional value is of great importance to consumers who care about their health and weight. However, it is affected by many variables such as feed, sex, breed, age, type of muscle, and method of production [36]. In the present study, we noticed that T2 significantly increased breast ash content, but T3 numerically improved dry matter, protein, and fat content of breast and leg muscles without reaching a significant level. This observation agrees with that of Sharmin et al. [17], who found no major effects of 1% and 1.5% of MOM on the meat composition of the thigh of broiler chickens raised in confinement. These authors reported similar results found in the current study for breast muscle proximate composition, except for fat content that was lower in birds fed the MOM diets. Comparably, Nkukwana et al. [37] observed that the breast nutrient content of mixed-sex broiler chicken intensively grown was not altered by the addition of MOM, but fat content was. Likewise, Tesfaye et al. [38] showed that the chemical composition of breast muscle from unsexed broiler chickens farmed indoors was unaffected by dietary MOM supplementation, but only the protein content of thigh muscle was significantly influenced by the treatments (0%, 5%, 10%, 15%, and 20% MOM). Simultaneously, Patel et al. [39] noticed that protein content increased in the breast and thigh muscles of colored chicken Chabro housed in a deep litter system with up to 5% MOM. However, Muis et al. [40] detected a major increase in protein content and an important decrease in fat content in meat from male broiler chickens fed with up to 15% of MOM powder. Furthermore, chicken meat’s moisture, lipid, and protein contents were statistically different with 0, 2, 4, and 8% of MOM powder in a study by Djouhou et al. [41], where the birds were reared in total confinement. Moreover, the work of Mickdam et al. [42] demonstrates that the addition of up to 7% MOM resulted in a significant increase in dry matter, protein, and ether extract in the breast and thigh of unsexed broilers intensively raised, while ash content was unaltered. The current experiment is reporting 11.34% and 6.4% more ash content in the breast and an additional 1.11% and 6.37% ash amount in the leg meat with the supplementation of T2 and T3, respectively. This increment in ash substance may be attributed to the moringa’s high mineral amount. As for differences between investigations, they might be due to the rate of MO inclusion, its chemical composition, the birds’ genotype sex and age, and the production or rearing system used in each study. Remarkably, none of the previously cited studies used outdoor access, which has important effects on the bird’s meat nutrient composition [4]. On the other hand, our study revealed that leg muscle had at least three times more fat content than breast meat. This result reinforced the fact that chicken breast and leg muscles differ in their chemical composition, and that the breast portion contains more protein but less fat than the leg muscle [43]. In a previous investigation, Evaris et al. [4] explained that the difference between these two types of muscles might be due to the traits of the fibers of each muscle type, as leg muscles have slow-twitch fibers that require fat as fuel but breast muscles (with fast-contraction fibers) use glycogen as the primary energy source, prompting less fat to be deposited in pectoral than in femoral muscles.

Regarding the fatty acid profile, oleic acid (C18:1N9C) was the most abundant in both breast and leg muscles. This report is similar to that of Nkukwana et al. [44], who found that chicken meat is rich in oleic acid. Moreover, several authors have reported that dietary MOM increased chicken meat’s omega-3 fatty acids [17,18,19]. On the other hand, stearic acid was the only fatty acid that numerically increased in both breast and leg muscles with MOM dietary supplementation. While palmitic acid, palmitoleic acid, oleic acid, and linoleic acid numerically decreased in breast muscle, all but linoleic acid increased in leg meat with MOM addition. This result implies that saturated fats decreased in the breast but increased in leg muscle. This observation, coupled with the presence of the polyunsaturated omega-6 fatty acid arachidonic, makes the breast meat healthier than the leg portion in this study. In fact, Žlender et al. [45] made the same observation when they reached the conclusion that chicken breast has a better fatty acid composition than leg muscle. According to Milićević et al. [46], differences in tissue fatty acid profile might be linked to the functions of the fatty acids in each tissue and to the tissue’s phospholipid content.

### 4.2. Influence of Moringa oleifera Lam. Meal (MOM) on Bone Quality

Macro-minerals such as calcium and phosphorus are essential for leg bone development as the femur and tibia support the live weight load of the bird [4]. Healthy bone is beneficial for both the producer and the animal as it is associated with economic loss and animal welfare. Indeed, Mutuş et al. [9] noted that nutritional factors, growth rate, and sex affect the bird’s normal bone development. In our study, the inclusion of MOM in the diet of slow-growing male chickens raised with outdoor access resulted in most parameters used to evaluate bone quality tending to record the lowest value with the T2 diet. This observation does not mean a completely negative impact at all. For instance, the least femoral and tibial robusticity index with T2 indicated that the long bones of the pelvic limb tended to be stronger and more resistant to fracture. However, the slightly higher value of the femoral and tibial Seedor index from birds fed T3 implied that these bones are inclined to be denser. Moreover, the higher amount of ash and phosphorous content (*P* ≤ 0.05) found in the bones of chickens fed with T3 revealed that these birds had better bone mineralization than the ones fed without MOM. It also indicated a proportionate body mass in relation to skeletal support [47]. This might be due to the moringa’s high amount of minerals, as stated by Rehman et al. [48]. The tibial diameter was considerably lower in birds fed with T1, whereas femoral and tibial lengths significantly decreased with the T2 diet. This observation implies that the use of T3 might have a better influence on bone quality. Generally, actual results show that MOM had no important effect (*P* > 0.05) on certain bone parameters such as bone calcium content, femoral and tibial weight, relative density, Seedor index, robusticity index, and femoral diameter. According to Salaam et al. [33], the absence of statistical significance for these bone parameters is a good indicator of a relatively balanced growth rate and bone development. The results of the present trial agree with those of Rehman et al. [48] for the influence of MO on ash content but disagree with their tibial weight and diameter, and robusticity index outcome. Similarly, our investigation is not in concordance with that of Nkukwana et al. [49], who found no effect of moringa on the tibial length and ash percentage. As previously mentioned, bone traits are not only influenced by diet but also by the growth rate, gender, environment, and production systems [4,8,9,10,11]. These might be the reasons for the discrepancies found between the present work and the previous studies reported in the literature, as those authors used fast-growing broiler chickens raised in total confinement.

## 5. Conclusions

Results from this study demonstrated that MOM tended to positively modify chicken meat chemical characteristics and clearly influenced the long bones of the pelvic limb of the birds when they had access to the outdoors. This is beneficial for the producer and animal as both can take advantage of this production system. Breast muscle and leg bone ash contents were higher with the supplementation of MOM, suggesting a higher mineral amount. The chemical composition of chicken breast muscle is different from that of leg muscle, as breast muscle seems to be healthier than the leg portion with the addition of MOM to the diet. This information is particularly useful to consumers who pay attention to their diet. Therefore, when added to the diet, up to 6% MOM does not have a detrimental effect on meat quality and may have beneficial impacts on the bone quality of slow-growing layer-type male chickens raised with outdoor access in the Mexican Tropic. Undoubtedly, further research is needed with slow-growing breeds and outdoor production systems as it is clear that results from broiler chickens and intensive production systems cannot be easily extrapolated.

## Figures and Tables

**Table 1:** Chemical analysis of *Moringa oleifera* Lam. meal.

Chemical Analysis	% Dry Matter
Crude Protein	19.11
Crude Fiber	15.39
Neutral Detergent Fiber	48.78
Acid Detergent Fiber	25.43
Ether Extract	2.97
Ash	8.74
Lignin	5.70
Condensed Tannins	3.27
Total Tannins	0.92
Total Phenols	2.78
**Fatty acid profile**	**g/100 g Fat**
C12–Lauric acid	0.921
C14–Myristic acid	2.733
C16–Palmitic acid	7.647
C16:1–Palmitoleic acid	1.223
C18:0–Stearic acid	4.987
C18:1n9T–Elaidic acid	3.980
C18:1n9C–Oleic acid	2.780
C18:2n6C–Linoleic acid	12.707
C20–Arachidic acid	59.182
C22:1n9–Eurucic acid	1.718
C24–Lignoceric acid	2.117

**Table 2:** Meat proximate composition of slow-growing layer-type male chickens fed with *Moringa oleifera* meal and reared with outdoor access in a tropical environment.

	*Moringa oleifera* Meal Level (%)			
	T1	T2	T3	*P*-Value
	(Mean ± SEM)	(Mean ± SEM)	(Mean ± SEM)	
**Breast muscle** ^1^ (%)				
Dry Matter	26.44 ± 0.27	26.68 ± 0.22	26.7 ± 0.50	0.847
Ash content	3.44 ^b^ ± 0.06	3.83 ^a^ ± 0.09	3.66 ^ab^ ± 0.05	0.005
Protein	23.6 ± 0.22	23.94 ± 0.22	24.09 ± 0.48	0.567
Fat	2.62 ± 0.16	2.19 ± 0.15	2.23 ± 0.24	0.228
**Leg muscle** ^2^ (%)				
Dry Matter	25.35 ± 0.24	25.83 ± 0.64	24.57 ± 0.43	0.193
Ash content	3.61 ± 0.08	3.65 ± 0.09	3.84 ± 0.06	0.106
Protein	21.58 ± 0.38	21.65 ± 0.81	21.63 ± 0.39	0.854
Fat	10.52 ± 0.88	10.39 ± 0.73	8.28 ± 1.05	0.173

^a,b^ Means with different letters are significantly different (*P* ≤ 0.05). SEM: standard error of mean. ^1^ breast muscle: 6 samples/treatment; ^2^ leg muscle: 6 samples/treatment. T1: 0% MOM; T2: 3% MOM; T3: 6% MOM.

**Table 3:** Fatty acid profile (g/100 g of fat) of breast and leg muscles of slow-growing layer-type Dominant Blue D 107 male chickens fed with *Moringa oleifera* meal and reared with outdoor access in a tropical climate.

	Dietary *Moringa oleifera* Meal Levels		
Traits	T1	T2	T3
**Breast muscle** ^1^			
Palmitic acid (C16)	29.79	29.40	28.77
Palmitoleic acid (C16:1)	2.67	2.12	1.72
Stearic acid (C18)	11.58	16.06	16.37
Oleic acid (C18:1N9C)	30.64	29.40	27.22
Linoleic acid (C18:2N6C)	21.60	19.36	20.31
Arachidonic acid (C20:4N6)	3.43	3.28	4.78
**Leg muscle** ^2^			
Palmitic acid (C16)	25.89	26.10	27.31
Palmitoleic acid (C16:1)	3.11	3.38	3.22
Stearic acid (C18)	12.44	12.51	14.15
Oleic acid (C18:1N9C)	33.24	35.17	33.45
Linoleic acid (C18:2N6C)	21.35	21.16	19.88

^1^ breast muscle: 1 composite sample/treatment; ^2^ leg muscle: 1 composite sample/treatment. T1: 0% MOM; T2: 3% MOM; T3: 6% MOM.

**Table 4:** Effects of *Moringa oleifera* meal on bone quality of slow-growing layer-type male chickens reared with outdoor access in a tropical atmosphere.

Traits	T1 (Mean ± SEM)	T2 (Mean ± SEM)	T3 (Mean ± SEM)	*P*-Value
Femoral Weight (g)	17.51 ± 0.26	16.93 ± 0.26	17.44 ± 0.27	0.249
Femoral Length (cm)	10.60 ^a^ ± 0.06	10.37 ^b^ ± 0.06	10.63 ^a^ ± 0.06	0.008
Femoal Seedor Index (g/cm)	1.64 ± 0.02	1.63 ± 0.08	1.65 ± 0.02	0.813
Femoral Robusticity Index (cm/g)	4.09 ± 0.02	4.05 ± 0.02	4.10 ± 0.02	0.163
Femoral Diameter (cm)	1.03 ± 0.01	1.03 ± 0.01	1.05 ± 00.01	0.495
Femoral Relative Density	0.74 ± 0.01	0.72 ± 0.01	0.74 ± 0.01	0.225
Tibial Weight (g)	24.85 ± 0.34	24.18 ± 0.34	25.01 ± 0.35	0.194
Tibial Length (cm)	15.40 ^a^ ± 0.10	14.98 ^b^ ± 0.10	15.37 ^a^ ± 0.11	0.009
Tibial Seedor Index (g/cm)	1.61 ± 0.02	1.61 ± 0.02	1.63 ± 0.02	0.814
Tibial Robusticity Index (cm/g)	5.28 ± 0.03	5.19 ± 0.03	5.19 ± 0.03	0.055
Tibial Diameter (cm)	0.96 ^b^ ± 0.01	1.01 ^a^ ± 0.01	1.01 ^a^ ± 0.01	0.021
Tibial Relative Density	1.06 ± 0.02	1.03 ± 0.02	1.07 ± 0.02	0.145
Leg Bones ^1^ Dry Matter ^2^ (%)	64.80 ± 0.94	66.84 ± 1.62	64.98 ± 1.07	0.451
Leg Bones Ash ^3^ (%)	35.01 ^b^ ± 0.44	35.68 ^ab^ ± 0.52	37.02 ^a^ ± 0.53	0.024
Leg Bones Calcium ^4^ (%)	6.03 ± 0.11	6.04 ± 0.19	6.48 ± 0.13	0.080
Leg Bones Phosphorus ^5^ (%)	6.64 ^b^ ± 0.15	6.91 ^ab^ ± 0.10	7.41 ^a^ ± 0.30	0.046

^a,b^ Means that do not share similar letters are significantly different (*P* ≤ 0.05). ^1^ Leg Bones: Femur + Tibia; ^2^ Dry Matter: 12 samples per treatment; ^3^ Ash content: 12 samples per treatment; ^4^ Calcium content: 6 samples per treatment; ^5^ Phosphorus content: 6 samples per treatment. T1: 0% MOM; T2: 3% MOM; T3: 6% MOM.

## Data Availability

Data presented in this study are available on fair request from the authors.
